# The Histone Demethylase JMJD1C Regulates CAMKK2-AMPK Signaling to Participate in Cardiac Hypertrophy

**DOI:** 10.3389/fphys.2020.00539

**Published:** 2020-06-18

**Authors:** Shuang Yu, Yihong Li, Hongwei Zhao, Qingdong Wang, Ping Chen

**Affiliations:** ^1^Department of Cardiology, First Affiliated Hospital of Jiamusi University, Jiamusi, China; ^2^Department of Emergency, First Affiliated Hospital of Jiamusi University, Jiamusi, China; ^3^Department of Anesthesiology, First Affiliated Hospital of Jiamusi University, Jiamusi, China; ^4^Department of Obstetrics and Gynecology, First Affiliated Hospital of Jiamusi University, Jiamusi, China

**Keywords:** cardiac hypertrophy, histone methylation, JMJD1C, AMPK, CAMKK2

## Abstract

The roles of the histone demethylase JMJD1C in cardiac hypertrophy remain unknown. JMJD1C was overexpressed in hypertrophic hearts of humans and mice, whereas the histone methylation was reduced. *Jmjd1c* knockdown repressed the angiotensin II (Ang II)-mediated increase in cardiomyocyte size and overexpression of hypertrophic genes in cardiomyocytes. By contrast, JMJD1C overexpression promoted the hypertrophic response of cardiomyocytes. Our further molecular mechanism study revealed that JMJD1C regulated AMP-dependent kinase (AMPK) in cardiomyocytes. JMJD1C did not influence LKB1 but repressed *Camkk2* expression in cardiomyocytes. Inhibition of CAMKK2 with STO609 blocked the effects of JMJD1C on AMPK. AMPK knockdown blocked the inhibitory functions of JMJD1C knockdown on Ang II-induced hypertrophic response, whereas metformin reduced the functions of JMJD1C and repressed the hypertrophic response in cardiomyocytes.

## Introduction

Currently, cardiovascular disease has become the leading cause of death all around the world. Cardiac hypertrophy is a pathological foundation of diverse cardiovascular diseases, including heart failure and hypertension (Veselka et al., 2017). The heart of mature mammals shows the low potential of cardiomyocyte proliferation. When the heart is injured and cardiomyocyte apoptosis occurs, the number of cardiomyocytes decreases (Nakamura and Sadoshima, 2018). The cardiomyocytes are unable to proliferate to support the increased demand of the heart. Instead, the cardiomyocytes undergo growth or hypertrophy to satisfy the demand. Pathological cardiac hypertrophy is a fundamental mechanism underlying diverse cardiovascular diseases, and sustained hypertrophy leads to arrhythmia and heart failure (Hou and Kang, 2012).

Metabolic dysfunction is a core mechanism underlying cardiac hypertrophy. Under physiological conditions, the cardiomyocytes use fatty acids to support the energy demand of the heart (Noordali et al., 2018). However, when pathological cardiac hypertrophy occurs, the cardiomyocytes undergo a metabolic switch (Riehle and Abel, 2016; Noordali et al., 2018). In hypertrophic cardiomyocytes, the cells prefer to utilize glucose as the energy source (Riehle and Abel, 2016). The metabolism of cardiomyocytes is regulated by several core regulators, including Sirtuins, AMPK, FoxO, and insulin signaling (Doenst et al., 2013; Riehle and Abel, 2016). For example, AMPK signaling is repressed during cardiac hypertrophy, and cardiac-specific knockout of AMPK promoted stress-induced cardiac hypertrophy (Salt and Hardie, 2017). By contrast, metformin-mediated activation of AMPK prevented metabolic dysfunction and cardiac hypertrophy as well as heart failure (Daskalopoulos et al., 2016). Generally, AMPK activation is induced by the calcium/calmodulin–dependent protein kinase (CAMKK2) or the AMP-dependent liver kinase B1 (LKB1) (Steinberg and Carling, 2019). Inactivation of either LKB1 or CAMKK2 could facilitate the development of metabolic dysfunction and cardiac hypertrophy (Dolinsky et al., 2009; Watanabe et al., 2014). However, the mechanism by which the AMPK signaling is regulated during cardiac hypertrophy is not fully understood.

In general, H3K4 (lysine 4 at histone H3) methylation is correlated with gene activation, whereas the methylation of H3K9 (H3K9me) and H3K27 (H3K27me) is related to the repression of gene transcription (Greer and Shi, 2012). The demethylation of H3K9 is generally controlled by the Jumonji C domain-containing proteins (JMJD), which include the PHF8, JMJD1 family, and JMJD2 family (Jambhekar et al., 2019). The histone demethylase JMJD1C can remove the methylation motif from H3K9me1, H3K9me2, and H3K9me3 (Michalak et al., 2019). JMJD1C and WHISTLE mediate the balance of histone methylation by WHISTLE and demethylation by JMJD1C with WHISTLE acting as a transcriptional repressor during testis development in mice (Kim et al., 2010). During DNA break repair, the histone demethylase JMJD1C targets MDC1 to regulate the response of chromatin to DNA breaks mediated by RNF8 and BRCA1 (Watanabe et al., 2013). JMJD1C also regulates the expression of miR-302 to inhibit the differentiation of human embryonic stem cells to neurons (Wang et al., 2014). In addition, JMJD1C ensures cellular self-renewal and reprogramming by controlling the expression of microRNAs in mouse embryonic stem cells (Xiao et al., 2017). JMJD1C is also required for the maintenance of leukemia. JMJD1C depletion induces a growth defect that is primarily attributed to the increase in apoptosis of leukemia cells from either mouse or human (Sroczynska et al., 2014). JMJD1C is critically involved in the survival of acute myeloid leukemia cells by serving as the coactivator of pivotal transcription factors (Chen et al., 2015). Furthermore, JMJD1C essentially contributes to MLL-AF9/HOXA9-mediated self-renewal of leukemia stem cells (Zhu et al., 2016). Partially, JMJD1C-induced metabolic dysfunction contributes to HOXA9-dependent leukemogenesis (Lynch et al., 2019), which implies that JMJD1C may be a regulator of cellular metabolism. The role of JMJD1C in the cardiovascular system is not known.

Here, we investigate the roles of JMJD1C during cardiac hypertrophy in humans and mice. JMJD1C was significantly overexpressed in hypertrophic hearts of humans and mice, which was coupled with the downregulation of the methylation of H3K9. JMJD1C promoted cardiomyocyte hypertrophy induced by Ang II, which relied on the AMPK signaling in a CAMKK2-dependent manner.

## Materials and Methods

### Heart Samples From Humans

Ten tissue samples of patients with hypertrophic cardiomyopathy and 10 from control donors were enrolled in this study. The hypertrophic heart tissues were obtained from patients who were diagnosed with hypertrophic cardiomyopathy (HCM). The control heart tissues were obtained from vehicle accident victims. The samples were obtained and stored in liquid nitrogen after informed consent was signed by the patients or the families of the donors. The study was approved by the institutional review boards for a clinical study at Jiamusi University.

### Animal Experiments

Cardiac hypertrophy in 8-week-old male C57BL/6N mice was induced by chronic infusion of angiotensin II (Sigma, 1.5 mg/kg/day) for a continuous 4 weeks as described previously (Tang et al., 2017). In brief, an Ang II-contained minipump (ALZET 2004) was placed into the mouse subcutaneously. Mice and SD rats were purchased from Charles River. The protocol for the animal study was approved by the ethics review boards at Jiamusi University.

### Isolation and Treatment of Cardiomyocyte

Cardiomyocytes were isolated from the heart ventricles of 1- to 3-day neonatal SD rats as described previously (Tang et al., 2016). Neonatal rat cardiomyocytes (NRCMs) were then cultured in DMEM medium supplied with 10% fetal bovine serum (FBS, Gibco), 1% antibiotic-antimycotic (ThermoFisher) for 48 h. The NRCMs were cultured in DMEM with 1% fetal bovine serum for 24 h and then the hypertrophy of NRCMs was induced by angiotensin II (1 μM) treatment for 48 h as reffed from previous publications (Luo et al., 2017; Tang et al., 2017). Cardiomyocyte size was measured with Image J software. For each group at each experiment, >100 cardiomyocytes were quantified for average cardiomyocyte size, and the results of three independent experiments were shown.

### Preparation of Adenovirus

For gene knockdown or overexpression, the adenovirus system was applied. Adenoviral vectors expressing rat *Jmjd1c* (Ad-*Jmjd1c*), control construct (Ad-Ctrl), sh*Jmjd1c* (Ad-sh*Jmjd1c*, shRNA sequence: GCGCTGACCTTCAAACCATTG), or sh*Prkaa1* (Ad-sh*Prkaa1*, shRNA sequence: GCACGAGTTGACTGGACATAA) and negative control (Ad-shCtrl) were obtained using the AdEasy Vector kit (Jia et al., 2014).

### Quantitative Real-Time PCR (qRT-PCR)

The cultured cells and heart tissues were washed with cold PBS and then subjected to RNA extraction with TRIZOL reagent (ThermoFisher). Next, the SuperScript III first-strand synthesis system kit (Invitrogen) was applied for cDNA synthesis with 1 ug of total RNA. Finally, SYBR green real-time PCR master mixes (Invitrogen) were applied to analyze the expression of target genes with the cDNA. The primers are shown in Table 1. The double delta CT method was applied for the quantification of mRNA expression.

**Table 1:** Primers used for quantitative real-time PCR.

**Gene name**	**Forward primer (5^**′**^-3^**′**^)**	**Reverse primer (5^**′**^-3^**′**^)**
Human *JMJD1C*	CAGGTCTCGTGCCAATCAAAA	GCTGTTGCTGGTGTGTATTCT
Human *Tubulin*	TGGACTCTGTTCGCTCAGGT	TGCCTCCTTCCGTACCACAT
Human *ANP*	GATGGTGACTTCCTCGCCTC	AAGAAAGCACACCAACGCAG
Human *BNP*	TGGAAACGTCCGGGTTACAG	CTGATCCGGTCCATCTTCCT
Human *MYH7*	AGTGGCAATAAAAGGGGTAGC	CCAAGTTCACTCACATCCATCA
Mouse *JMJD1C*	CACCCGCACCATGATCGTTAT	CTTCGCCGTGATGTAATGCC
Mouse *Tubulin*	CACTTACCACGGAGATAGCGA	ACCTTCTGTGTAGTGCCCCTT
Mouse *ANP*	GCTTCCAGGCCATATTGGAG	GGGGGCATGACCTCATCTT
Mouse *BNP*	GAGGTCACTCCTATCCTCTGG	GCCATTTCCTCCGACTTTTCTC
Mouse *MYH7*	CATGGGATGGTAAGAAACGGG	TCCTCCAGTAAGTCGAAACGG
Rat *JMJD1C*	AGCTAGTGGGAAAGCGGTTC	AATTCCACGTAGACCGCCAG
Rat *Tubulin*	CAACTATGTGGGGGACTCGG	TGGCTCTGGGCACATACTTG
Rat *ANP*	CCTGGACTGGGGAAGTCAAC	ATCTATCGGAGGGGTCCCAG
Rat *BNP*	TGACGGGCTGAGGTTGTTTT	ACACTGTGGCAAGTTTGTGC
Rat *MYH7*	CCCAACCCTAAGGATGCCTG	TGTGTTTCTGCCTAAGGTGCT
Rat *CAMKK2*	AGAACTGCACACTGGTCGAG	CCGGCTACCTTCAAATGGGT

### Western Blot

The cultured cells and heart tissues were washed with cold PBS and then subjected to protein extraction with RIPA buffer (Millipore) supplemented with a phosphatase inhibitor cocktail (Roche) and a protease inhibitor cocktail (Roche). Protein concentration was measured with a BCA kit (ThermoFisher). SDS-PAGE was applied to separate the proteins in gel with 30 ug of total protein; then the proteins were transferred to PVDF membranes (Beyotime) followed by blockade with 5% fat-free milk in TBST for 1 h. Then, the membranes were washed with TBST three times and incubated with primary antibodies at 4°C overnight. The next day, the membranes were washed and incubated with HRP-conjected secondary antibodies. Finally, the expression of proteins was analyzed with the chemiluminescent Western blot detection kit (Millipore). The following primary antibodies were used: anti-Tubulin antibody (Cell Signaling Technology, #2144), anti-JMJD1C antibody (ThermoFisher, #PA5-20804), anti-Histone H3 antibody (Abcam, #ab1791), anti-H3K9me1 antibody (Active Motif, #39249), anti-H3K9me2 antibody (Active Motif, #39683), anti-H3K9me3 antibody (Active Motif, #61013), anti-AMPK antibody (Proteintech, #66536-1-Ig), anti-pAMPK antibody (Cell Signaling Technology, #2535), anti-ACC antibody (Proteintech, #67373-1-Ig), anti-pACC antibody (Cell Signaling Technology, #11818), anti-LKB1 antibody (Santa Cruz Biotechnology, #sc-32245), anti-pLKB1 antibody (Santa Cruz Biotechnology, #sc-271924), anti-CAMKK2 antibody (ThermoFisher, #PA5-69921), anti-PGC1a antibody (Cell Signaling Technology, #2178), anti-O-GlcNAcylation antibody (Abcam, #ab2739), anti-p-p70S6K1 antibody (Cell Signaling Technology, #9205), anti-p70S6K1 antibody (Cell Signaling Technology, #2708), anti-p-eEF2 antibody (Cell Signaling Technology, #2331), anti-eEF2 antibody (Cell Signaling Technology, #2332), anti-H3K4me1 antibody (Abcam, #ab8895), anti-H3K4me3 antibody (Abcam, #ab8580), anti-H3K9ac antibody (Active Motif, #61663).

### Chromatin Immunoprecipitation (ChIP) Assay

A ChIP assay was performed with the chromatin immunoprecipitation (ChIP) assay kit (17-295) with ChIP-grade primary antibodies. Immunoprecipitated chromatin fragments were quantified by SYBR-based qRT-PCR, normalized using the input.

### Protein Synthesis Assay

The protein synthesis assay was performed by analyzing the incorporation of [^14^C]-phenylalanine into proteins of cultured NRVMs as described previously (Sundaresan et al., 2009).

### Statistical Analysis

The statistical analysis was performed with SPSS software, and all the values are expressed as mean ± *SD*. Student's *t-*test was applied for the analysis of the difference between the two groups. One-way ANOVA followed by Tukey's *post hoc* test was used when more than two groups exist.

## Results

### JMJD1C Expression and Histone H3K9 Methylation Levels Were Changed in Hypertrophic Hearts in Human and Mice

**Figure 1:**
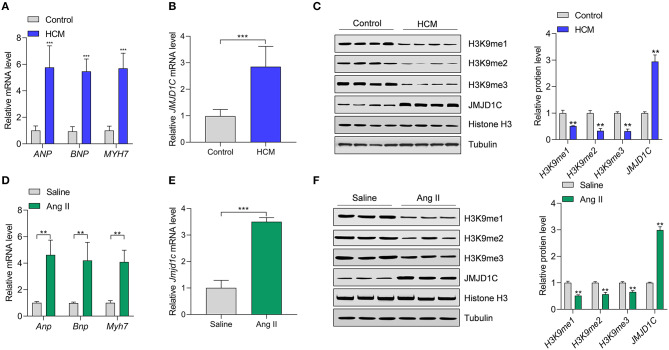
JMJD1C expression is increased in hypertrophic hearts. **(A)** Expression of hypertrophy-related fetal genes (*ANP, BNP, MYH7*) in hypertrophic hearts (HCM) of human patients (*n* = 10) and in control donors (*n* = 10). ***p < 0.001 vs. control. **(B)** JMJD1C mRNA expression in hypertrophic hearts of human patients (*n* = 10) and in control donors (*n* = 10). ***p < 0.001 vs. control. **(C)** JMJD1C protein and levels of related histone methylation marks (H3K9me1, H3K9me2, H3K9me3) in hypertrophic hearts of human patients and in control donors. **p < 0.01 vs. control. *n* = 4 in each group. **(D)** Hypertrophy-related fetal gene levels in the hearts of mice undergoing saline or Angiotensin II (Ang II) infusion for 4 weeks. **p < 0.01 vs. saline. *n* = 5 in each group. **(E)** *Jmjd1c* mRNA expression in control and hypertrophic hearts of mice treated as in **(D)**. ***p < 0.001 vs. saline. *n* = 5 in each group. **(F)** Levels of JMJD1C protein and related histone methylation marks (H3K9me1, H3K9me2, H3K9me3) in the control and hypertrophic hearts of mice treated as in **(D)**. In **(C,F)**, a representative tubulin blot is shown as control, and all the loading controls were very similar. **p < 0.01 vs. saline. *n* = 4 in each group.

To understand the functions of JMJD1C in cardiac hypertrophy, JMJD1C expression was examined in hypertrophic hearts of humans and mice. We collected heart tissues from 10 cases of hypertrophic cardiomyopathy (HCM) and from 10 control donors. We confirmed the upregulation of hypertrophy-related fetal genes (e.g., *ANP, BNP*, and *MYH7*) in HCM tissues compared with the control donors (Figure 1A). Compared with control tissues, the expression levels of JMJD1C mRNA and protein were significantly increased in HCM tissues (Figures 1B,C). Next, we observed that the levels of H3K9me1, H3Kme2, and H3K9me3 were downregulated in HCM tissues, which was associated with the upregulation of JMJD1C (Figure 1C). In addition, cardiac hypertrophy was induced in mice by chronic infusion of Ang II for 4 weeks. The overexpression of hypertrophy-related fetal genes was confirmed in hypertrophic hearts (Figure 1D). In hypertrophic hearts, the expression of JMJD1C was remarkably increased (Figures 1E,F), and the levels of H3K9me1, H3K9me2, and H3K9me3 were decreased (Figure 1F). In addition, we observed the increase in methylation of H3K4 (H3K4me1 and H3K4me3) as well as acetylation of H3K9 in hypertrophic mouse hearts (Supplementary Figure 1). Collectively, these findings demonstrated that JMJD1C was overexpressed in cardiac hypertrophy, which was associated with the downregulation of methylation of H3K9.

### JMJD1C Controlled Cardiomyocyte Hypertrophy

**Figure 2:**
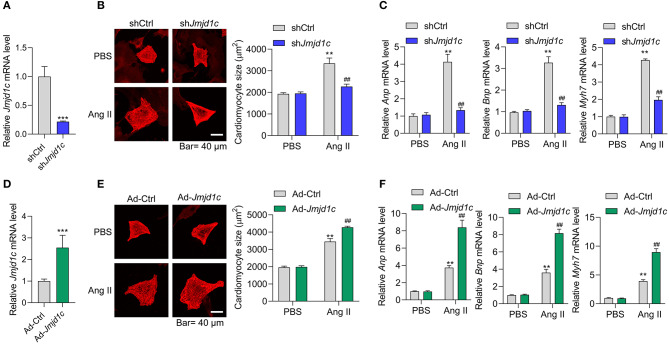
JMJD1C promotes cardiomyocyte hypertrophy. **(A)** *Jmjd1c* knockdown in neonatal rat cardiomyocytes (NRCMs). The cells were infected with adenovirus expressing sh*Jmjd1c* or shCtrl for 48 h. ***p < 0.001 vs. shCtrl. **(B,C)** *Jmjd1c* knockdown reduces the Ang II-mediated increase in the size of cardiomyocytes **(B)** and the expression of fetal genes **(C)**. Cardiomyocytes infected with adenovirus expressing sh*Jmjd1c* or shCtrl were treated with Ang II (1 μM) for 48 h to induce hypertrophy. Then α-actinin staining was performed and cardiomyocyte size was analyzed. **p < 0.01 vs. shCtrl+PBS, ^##^*p* < 0.01 vs. shCtrl+Ang II. **(D)** *Exogenous Jmjd1c* overexpression in NRCMs. The cells were infected with adenovirus overexpressing *Jmjd1c* for 48 h. ***p < 0.001 vs. Ad-Ctrl. **(E,F)** Exogenous *Jmjd1c* overexpression enhances the increase in cardiomyocyte size **(E)** and hypertrophic fetal gene expression **(F)** induced by Ang II. NRCMs with/without *Jmjd1c* overexpression were treated with Ang II for 48 h to induce hypertrophy. Then cardiomyocyte size was analyzed. **p < 0.01 vs. Ad-Ctrl+PBS, ^##^*p* < 0.01 vs. Ad-Ctrl+Ang II. All results are from three independent experiments.

**Figure 3:**
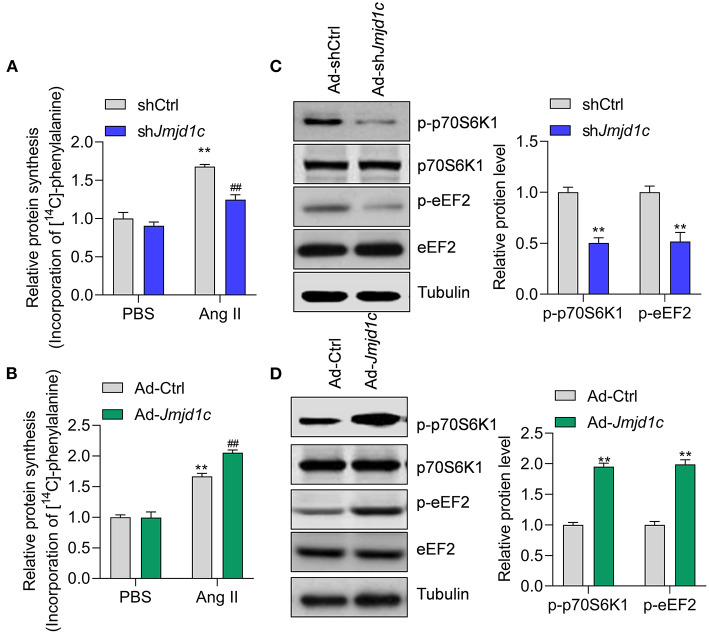
JMJD1C regulates protein synthesis in cardiomyocytes. **(A)** JMJD1C knockdown reduces Ang II-induced protein synthesis. **p < 0.01 vs. Ad-shCtrl+PBS; ^##^*p* < 0.01 vs. Ad-shCtrl+Ang II. *n* = 3 in each group. **(B)** JMJD1C overexpression promotes Ang II-induced protein synthesis. **p < 0.01 vs. Ad-Ctrl+PBS; ^##^*p* < 0.01 vs. Ad-Ctrl+Ang II. *n* = 3 in each group. **(C)** JMJD1C knockdown reduces phosphorylation of p70S6K1 and eEF2 in Ang II-treated cardiomyocytes. **p < 0.01 vs. Ad-shCtrl. *n* = 3 in each group. **(D)** JMJD1C overexpression increases phosphorylation of p70S6K1 and eEF2 in Ang II-treated cardiomyocytes. **p < 0.01 vs. Ad-Ctrl. *n* = 3 in each group.

Next, we investigated the roles of JMJD1C in the regulation of cardiac hypertrophy using neonatal rat cardiomyocyte (NRCMs). We knocked down the expression of *Jmjd1c* in NRCMs with adenovirus-mediated shRNA (Figure 2A) and induced cardiomyocyte hypertrophy with Ang II. Treatment with Ang II increased the size of cardiomyocytes and led to overexpression of the hypertrophy-related fetal genes *Anp, Bnp*, and *Myh7*. Knockdown of *Jmjd1c* repressed hypertrophy of cardiomyocytes induced by Ang II treatment (Figures 2B,C). We also overexpressed Jmjd1c in NRCMs with adenovirus-mediated overexpression (Figure 2D). *Jmjd1c* overexpression promoted the Ang II-mediated increase in the size of cardiomyocytes and the overexpression of hypertrophy-related fetal markers (Figures 2E,F). Protein synthesis is a hallmark of cardiac hypertrophy (Tang et al., 2020a); thus, we tested the effects of JMJD1C on protein synthesis by performing [^14^C]-phenylalanine incorporation assay. We observed that JMJD1C knockdown reduced Ang II-induced protein synthesis (Figure 3A), whereas JMJD1C overexpression promoted Ang II-induced protein synthesis (Figure 3B). In addition, we tested the effects of JMJD1C on proteins that critically participate in protein synthesis. We analyzed the phosphorylations of p70S6K1 and eEF2. The results showed that JMJD1C knockdown inhibited the phosphorylations of p70S6K1 and Eef2, whereas the opposite effects were observed in JMJD1C-overexpressed cardiomyocytes (Figures 3C,D). Taken together, these findings revealed that JMJD1C promotes cardiomyocyte hypertrophy partially via regulating protein synthesis.

### JMJD1C Regulates the Activation of AMPK

**Figure 4:**
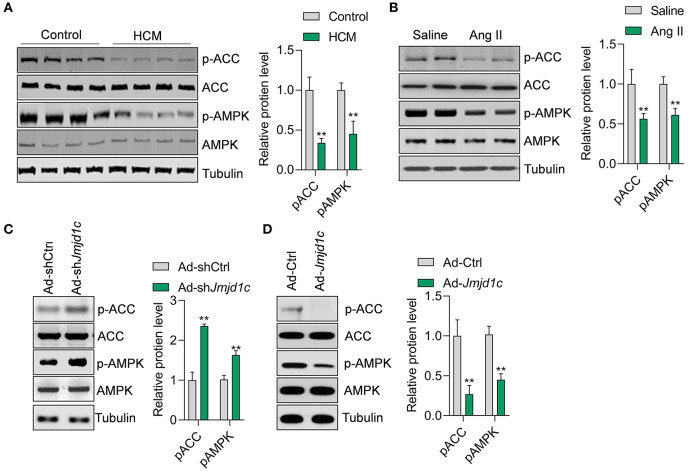
JMJD1C represses AMPK activation in cardiomyocytes. **(A,B)** The AMPK signaling pathway is repressed in hypertrophic hearts in humans and mice. **p < 0.01 vs. control or saline. *n* = 4 in each group. **(C)** *Jmjd1c* knockdown activates AMPK signaling. NRCMs were infected with adenovirus expressing sh*Jmjd1c* or shCtrl for 24 h, followed by Ang II treatment for an additional 24 h. **p < 0.01 vs. Ad-shCtrl. *n* = 3 in each group. **(D)** JMJD1C overexpression represses AMPK activation. NRCMs were infected with adenovirus expressing *Jmjd1c* for 24 h, followed by Ang II treatment for an additional 24 h. **p < 0.01 vs. Ad-Ctrl. *n* = 3 in each group.

We next explored the mechanism of JMJD1C function in hypertrophy of cardiomyocytes. Metabolism is critically involved in the physiological and pathological progress of the heart (Tang et al., 2020b). The metabolic sensor AMPK serves as one of the key regulators of glucose and fatty acid metabolism of cardiomyocytes, and the deregulation of AMPK signaling participates in hypertrophic cardiomyopathy and other cardiac diseases. In this study, we observed that the phosphorylation levels of the kinase AMPK and its downstream factor ACC were significantly downregulated in hypertrophic hearts in humans and mice (Figures 4A,B). Additionally, we observed that *Jmjd1c* knockdown promoted the activation of AMPK signaling, whereas *Jmjd1c* overexpression repressed AMPK signaling activation in Ang II-treated NRCMs (Figures 4C,D). AMPK was reported to regulate cardiac hypertrophy by inducing PGC1a (Peroxisome proliferator–activated receptor gamma coactivator 1-alpha) expression and reducing protein O-GlcNAcylation (Watanabe et al., 2014; Gelinas et al., 2018). Indeed, we observed that *Jmjd1c* knockdown increased the PGC1a protein level and reduced total protein O-GlcNAcylation, which was blocked by AMPK knockdown (Supplementary Figure 2). Therefore, these findings support the notion that JMJD1C represses AMPK signaling during cardiac hypertrophy.

### JMJD1C Regulates the Expression of CAMKK2 to Target AMPK

**Figure 5:**
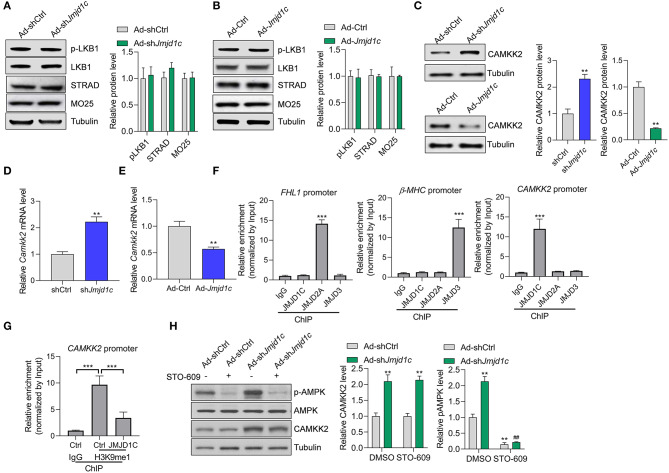
JMJD1C regulates AMPK in a CAMKK2-dependent manner. **(A,B)** JMJD1C does not affect activation of LKB1 or levels of its activation partners STRAD and MO25 in NRCMs. The cells were infected with adenovirus expressing *Jmjd1c*, sh*Jmjd1c*, or the control constructs for 24 h, followed by Ang II treatment for an additional 24 h. *n* = 3 in each group. **(C)** Effects of JMJD1C on CAMKK2 expression. NRCMs were treated as in **(A)**. **p < 0.01 vs. Ad-Ctrl or shCtrl. *n* = 3 in each group. **(D,E)** Effects of JMJD1C on CAMKK2 mRNA expression. NRCMs were treated as in **(A)**. **p < 0.01 vs. Ad-Ctrl or shCtrl. **(F)** Chromatin immunoprecipitation (ChIP) assay showing the binding of JMJD1C, JMJD2A, and JMJD3 on the FHL1, β-MHC, and CAMKK2 promoters. IgG was used for negative control, and the enrichment was normalized to the input. ***p < 0.001 vs. IgG. **(G)** ChIP assay showing that JMJD1C overexpression reduced H3K9me1 enrichment at the CAMKK2 promoter in cardiomyocytes. ***p < 0.001 **(H)**. The inhibition of CAMKK2 blocks the effects of JMJD1C on AMPK activation in NRCMs. The cells were treated with adenovirus expressing sh*Jmjd1c* or shCtrl; then the cells were treated with Ang II and the CAMKK2 inhibitor STO-609 (20 μM) for an additional 24 h. All results are from three independent experiments. **p < 0.01 vs. Ad-shCtrl+PBS; ^##^*p* < 0.01 vs. Ad-shCtrl+STO-609. *n* = 3 in each group.

We attempted to investigate the molecular mechanism underlying JMJD1C-mediated AMPK inactivation. AMPK is generally activated by two kinases, LKB1 and CAMKK2. We observed that LKB1 expression and activation and its interaction partners STRAD and MO25 were not affected by *Jmjd1c* knockdown or overexpression (Figures 5A,B). Interestingly, we observed that *Jmjd1c* knockdown increased the protein levels of CAMKK2, whereas *Jmjc1c* overexpression downregulated the protein levels of CAMKK2 in NRCMs (Figure 5C). Importantly, we showed that JMJD1C controlled the expression of CAMKK2 at the transcriptional level because JMJD1C overexpression reduced and knockdown increased the levels of CAMKK2 mRNA (Figure 5E). Next, we analyzed the mechanism by which JMJD1C regulated CAMKK2 expression. JMJD2A and JMJD3 were reported to regulate cardiac hypertrophy by epigenetically targeting the *FHL1 (four-and-a-half LIM domains 1)* and the β*-MHC* promoter, respectively (Zhang et al., 2011; Guo et al., 2018). Our chromatin immunoprecipitation assay did not reveal the enrichment of JMJD2A and JMJD3 at the CAMKK2 promoter nor of JMJD1C at the FHL1 or MHC promoters. By contrast, JMJD1C selectively bound the CAMKK2 promoter (Figure 5F). In addition, overexpression of JMJD1C reduced H3K9me1/2/3 levels at the CAMKK2 promoter (Figure 5G, Supplementary Figure 3). Finally, we treated NRCMs with the CAMKK2 inhibitor STO-609 and observed that STO-609 treatment blocked the roles of JMJD1C on the activation of the AMPK pathway (Figure 5H). These results demonstrated that JMJD1C controls AMPK signaling via a CAMKK2-dependent mechanism.

### AMPK Is Essentially Involved in the Function of JMJD1C

**Figure 6:**
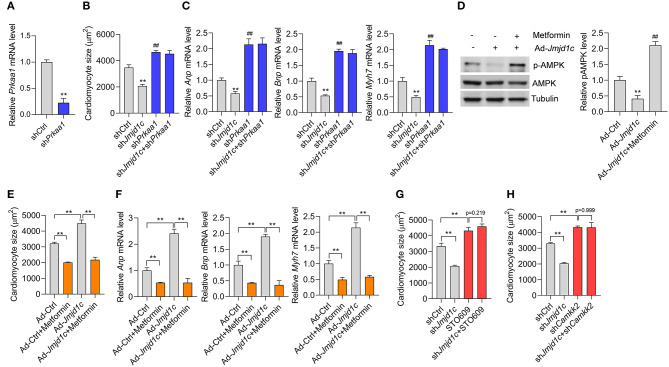
AMPK participates in the role of JMJD1C during cardiomyocyte hypertrophy. **(A)** *Prkaa1* knockdown in cardiomyocytes. The cells were treated with adenovirus expressing sh*Prkaa1* or with a control virus for 48 h **(B,C)**. *Prkaa1* knockdown blocks the influence of *Jmjd1c* knockdown on Ang II-induced increase in cardiomyocytes. The cells with/without *Prkaa1* or *Jmjd1c* knockdown were subjected to hypertrophy induction with Ang II for 48 h. Then cardiomyocyte size **(B)** and gene expression **(C)** were analyzed. **p < 0.01 vs. shCtrl. ##*p* < 0.01 vs. shCtrl **(D)**. Metformin activates AMPK. The indicated cardiomyocytes were treated with metformin (1 mM) for 24 h. **p < 0.01 vs. Ad-Ctrl; ^##^*p* < 0.01 vs. Ad-Jmjd1c. *n* = 3 in each group **(E,F)**. Metformin-mediated activation of AMPK blocks the influence of JMJD1C overexpression on cardiomyocyte hypertrophy induced by Ang II stimuli. NRCMs were infected with/without adenovirus carrying *Jmjd1c* for 24 h; then the cells were treated with Ang II (1 μM) metformin (1 mM) for an additional 48 h. Cardiomyocyte size **(E)** and gene expression **(F)** were analyzed **p < 0.01. **(G)** STO-609 blocks the influence of *Jmjd1c* knockdown on the Ang II-induced increase in cardiomyocytes. **p < 0.01. **(H)** *Camkk2* knockdown blocks the influence of *Jmjd1c* knockdown on Ang II-induced increase in cardiomyocytes. **p < 0.01. All results are from three independent experiments.

Finally, we investigated whether JMJD1C-mediated AMPK inactivation was critically involved in the function of JMJD1C. We knocked down the expression of AMPK (*Prkaa1*) in NRCMs (Figure 6A) and induced cardiomyocyte hypertrophy with Ang II. We observed that AMPK knockdown promoted the hypertrophic response induced by Ang II and blocked the prevention of cardiomyocyte hypertrophy mediated by *Jmjd1c* knockdown (Figures 6B,C). In addition, we observed that metformin, an AMPK activator, rescued AMPK activation in NRCMs with Jmjd1c overexpression (Figure 6D). Significantly, metformin treatment repressed cardiomyocyte hypertrophy and blocked the function of JMJD1C overexpression (Figures 6E,F). Finally, we also tested whether JMJD1C promoted cardiomyocyte hypertrophy via CAMKK2. We inhibited CAMKK2 with STO-609 or knocked down Camkk2 expression and observed that either inhibition of CAMKK2 with STO-609 or Camkk2 knockdown blocked the inhibitory effects of Jmjd1c knockdown on cardiomyocyte hypertrophy (Figures 6G,H). Therefore, AMPK signaling critically participated in the roles of JMJD1C during the hypertrophic response of cardiomyocytes.

## Discussion

Here we demonstrate that JMJD1C serves as an epigenetic regulator that is involved in pathological cardiac hypertrophy. The expression of JMJD1C mRNA and protein was increased during cardiac hypertrophy in humans and mice, which was associated with decreased methylation of H3K9. Further experimental evidence demonstrated that JMJD1C promoted the hypertrophic response of cardiomyocytes induced by Ang II. JMJD1C repressed AMPK through transcriptionally repressing the expression of CAMKK2 but not LKB1 in cardiomyocytes, which was involved in the roles of JMJD1C during cardiomyocyte hypertrophy.

Epigenetic regulators have been reported to be essential for the initiation and progress of cardiac hypertrophy. For instance, the histone deacetylase (HDACs) and Sirtuins participated in cardiac hypertrophy by targeting histone and non-histone targets (Li et al., 2019). The histone demethylase family is also critically involved in pathological cardiac hypertrophy. For instance, JMJD2A demethylated H3K9me3 and activated transcription of pro-hypertrophic genes. In animals, JMJD2A promoted cardiac hypertrophy (Zhang et al., 2011). KDM3A promoted pressure overload–induced cardiac hypertrophy and fibrosis. And KDM3A activated *Timp1* expression with pro-fibrotic activity in cardiomyocytes. Interestingly, JIB-04, the pan-KDM inhibitor, repressed pathological cardiac hypertrophy and fibrosis (Zhang et al., 2018).

In this study, JMJD1C expression levels were increased in hypertrophic hearts in mice and humans. The upregulation of JMJD1C was associated with downregulated levels of H3K9me1, H3K9me2, and H3K9me3. We knocked down or overexpressed JMJD1C in cardiomyocytes and found that JMJD1C promoted the increase in the size of cardiomyocytes and expression of hypertrophy-related fetal genes, including *Anp, Bnp*, and *Myh7*, during Ang II-induced cardiomyocyte hypertrophy. Therefore, the JMJD family members are generally pro-hypertrophic epigenetic regulators (Zhang et al., 2011, 2018). However, our study was based on cardiomyocytes *in vitro*; further study is needed to explore the *in vivo* function of JMJD1C during cardiac hypertrophy with a cardiomyocyte-specific JMJD1C knockout mouse line.

The metabolic switch is a hallmark of hypertrophic cardiomyocytes. The metabolic sensor AMPK has critical functions in cardiac hypertrophy by diverse metabolism-dependent and independent mechanisms (Daskalopoulos et al., 2016). The previous study implicated the histone deacetylase SIRT2 in the activation of AMPK via LKB1 to repress cardiac hypertrophy (Tang et al., 2017). However, the negative epigenetic regulator of AMPK during cardiac hypertrophy remains unknown. Here we observed that JMJD1C repressed the activation of AMPK during cardiac hypertrophy. However, JMJD1C-mediated repression of AMPK signaling did not depend on LKB1, but rather on reducing CAMKK2 expression. Finally, our findings showed that AMPK activation with metformin blocked the effects of JMJD1C hyperexpression, whereas AMPK knockdown blockaded the effects of JMJD1C downregulation during cardiac hypertrophy. These findings demonstrated that the AMPK signaling pathway is essentially involved in JMJD1C-mediated function in pathological cardiac hypertrophy.

JMJD2A and JMJD3 were reported to regulate cardiac hypertrophy by epigenetically targeting FHL1 and MHC promoter, respectively (Zhang et al., 2011; Guo et al., 2018). We observed that JMJD2A was enriched at the FHL1 and JMJD3 at the beta-MHC promoter, respectively, but neither bound to the CaMKK2 promoter. In contrast, JMJD1C bound to the CaMKK2 promoter and regulated methylation of H3K9me1, implicating JMJD1C in the regulation of the expression of CaMKK2 via demethylating H3K9. In this study, we observed that JMJD1C bound the CaMKK2 promoter and reduced the H3K9me level, which was associated with CaMKK2 gene silence. This finding was contradictory to previous notions that H3K9me was a mark of gene repression and that JMJD1C was a transcriptional activator (Chen et al., 2015). Thus, the effects of JMJD1C on CaMKK2 expression may also rely on other mechanisms. For instance, the JMJD1C-mediated changes in the local chromatin 3-D structure at the CaMKK2 promoter may also recruit some transcriptional repressor. The transcriptional repressive roles were also observed in JMJD2A (Mallette and Richard, 2012; Li et al., 2014). In leukemia cells, Runx1 (Runt-related transcription factor 1) directly interacts with and recruits JMJD1C to target genes (Chen et al., 2015). In addition, JMJD1C regulates Runx1 target genes by maintaining low H3K9me1/2 levels (Chen et al., 2015). Interestingly, the promoter and 5'UTR region of the human CaMKK2 gene has consensus DNA-binding sequences for Runx1 (Racioppi and Means, 2012). Given that Runx1 also plays a critical role in the cardiovascular system (McCarroll et al., 2018), it will be very interesting to investigate whether Runx1 is also enriched at the CAMKK2 promoter. To this end, JMJD1C regulated CaMKK2 expression via a complex mechanism. In addition, we did not observe the effects of JMJD1C on total H3K27me3 (data not shown). As such, JMJD2A, JMJD3, and JMJD1C may show different enrichment at the genome and regulate the expression of different genes.

Taken together, here we identified JMJD1C as a new epigenetic regulator of cardiac hypertrophy by targeting the metabolic AMPK pathway.

## Data Availability Statement

The datasets generated for this study are available on request to the corresponding author.

## Ethics Statement

The studies involving human participants were reviewed and approved by institutional review boards for a clinical study at Jiamusi University. The patients/participants provided their written informed consent to participate in this study. The animal study was reviewed and approved by ethics review boards at Jiamusi University.

## Author Contributions

SY and PC designed the study. SY and YL performed most of the experiments. PC wrote the manuscript. QW performed cell culture and qPCR experiments. All authors contributed to the article and approved the submitted version.

## Conflict of Interest

The authors declare that the research was conducted in the absence of any commercial or financial relationships that could be construed as a potential conflict of interest.
