# Supplementary material for: The Histone Demethylase JMJD1C Regulates CAMKK2-AMPK Signaling to Participate in Cardiac Hypertrophy
Source: Front Physiol. 2020 Jun 18;11:539. doi: 10.3389/fphys.2020.00539 (PMC7314990; doi:10.3389/fphys.2020.00539)
Supplement: Supplementary file 1 [file Table_1.docx]

**
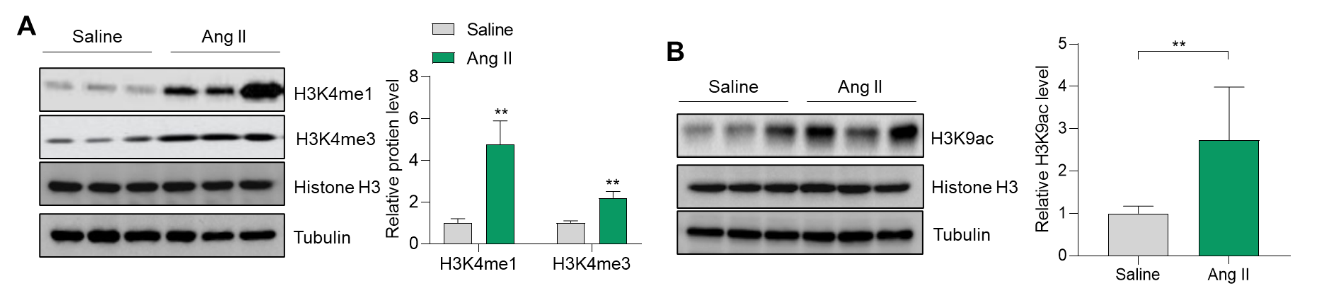
**

**Supplementary Figure 1 Methylation of H3K4 and acetylation of H3K9 in mouse hypertrophic hearts.** **p<0.01 vs. Saline. n=3 in each group.

**
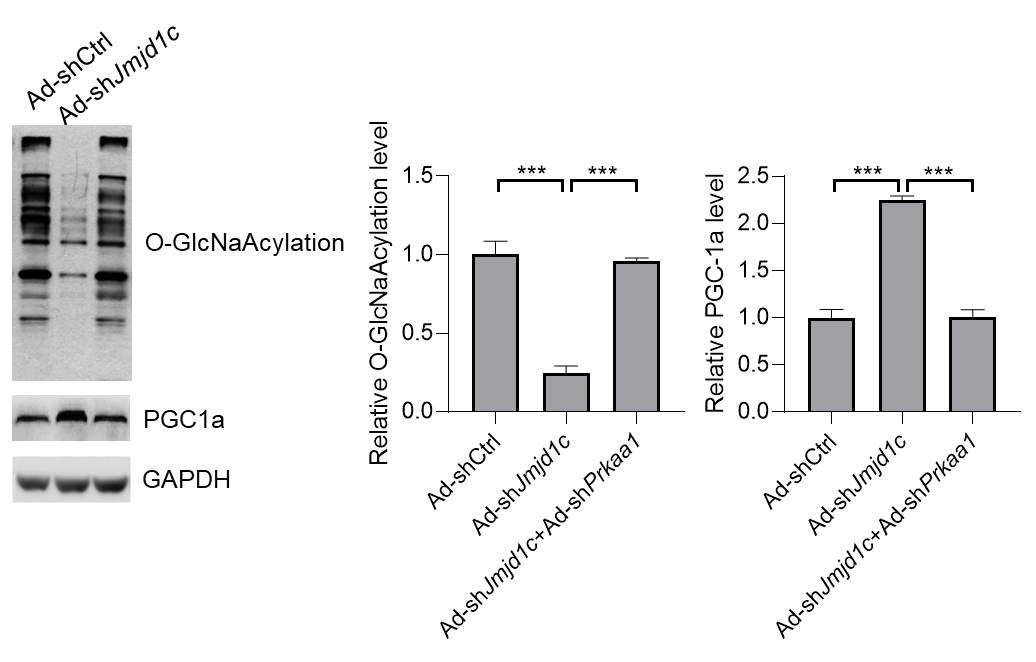
**

**Supplementary Figure 2 Effects of JMJD1C knockdown and AMPK (Prkaa1) knockdown regulates PGC1a expression and protein O-GlcNAcylation in cardiomyocytes.** ***p<0.001.

**
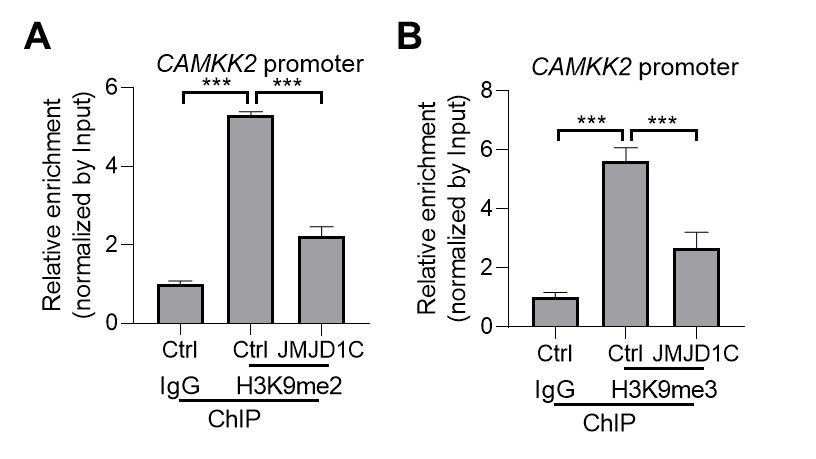
**

**Supplementary Figure 3 Effects of JMJD1C overexpression on H3K9me2/3 enrichment on *CAMKK2* promoter in cardiomyocytes.** ***p<0.001.
